# Trousseau Syndrome-Associated Acute Multifocal Cerebral Infarction: When Triple-Pathway Antithrombotic Therapy Overcomes Low Molecular Weight Heparin Resistance

**DOI:** 10.7759/cureus.80534

**Published:** 2025-03-13

**Authors:** Ke Wu, Lei Sheng

**Affiliations:** 1 Department of Neurology, The Second Affiliated Hospital of Nanjing University of Chinese Medicine, Nanjing, CHN

**Keywords:** acute multifocal cerebral infarction, anticoagulation, antiplatelet, low-molecular-weight heparin resistance, stroke, triple-pathway antithrombotic therapy, trousseau syndrome

## Abstract

Trousseau syndrome (TS), a malignancy-associated hypercoagulable state characterized by both arterial and venous synchronous thrombotic events, represents a critical clinical challenge. We present a 63-year-old male with hepatocellular carcinoma metastases who presented to the Emergency Department with acute-onset unconsciousness. Neurological evaluation revealed rare documentation of extensive multifocal cerebral infarcts accompanied by diffuse vascular thrombosis, atherosclerotic plaque burden, and markedly elevated D-dimer levels, establishing the diagnosis of TS. The initial infusion of nadroparin, which was co-administered with aspirin, achieved almost no anticoagulation. Consequently, the strategy was escalated to triple-pathway antithrombotic therapy through substitution with argatroban, clopidogrel, and cilostazol. This regimen successfully prevented further thrombotic complications. This case provides rare documentation of catastrophic cerebral parenchymal involvement secondary to TS. Furthermore, the effective application of anticoagulation-antiplatelet combination therapy following low-molecular-weight heparin (LMWH) resistance offers a potential management paradigm for refractory hypercoagulable states.

## Introduction

TS is a complex systemic coagulopathy characterized by arterial and venous thromboembolism associated with malignancy, whose pathogenesis remains unclear, but it is widely accepted that malignant tumors initiate a synergistic interaction of multiple mechanisms, such as tissue factor and cancer procoagulants along with other inflammatory cytokines, which together lead to a hypercoagulable state or disseminated intravascular coagulation [1]. For its rapid progression, frequent recurrence, and mortality rates second only to cancer itself, this paraneoplastic syndrome necessitates prompt recognition and tailored antithrombotic management [2]. We present a patient who presented with TS-associated acute multifocal cerebral infarction that was managed with triple-pathway antithrombotic therapy following low-molecular-weight heparin (LMWH) resistance, potentially attributed to plasma antithrombin deficiency. By sharing diagnosis and treatment, we aim to highlight the rare documentation of extensive multifocal cerebral lesions in TS and the triple-pathway anticoagulation-antiplatelet salvage therapy as a potential salvage approach to LMWH resistance cases.

## Case presentation

**Figure 1:**
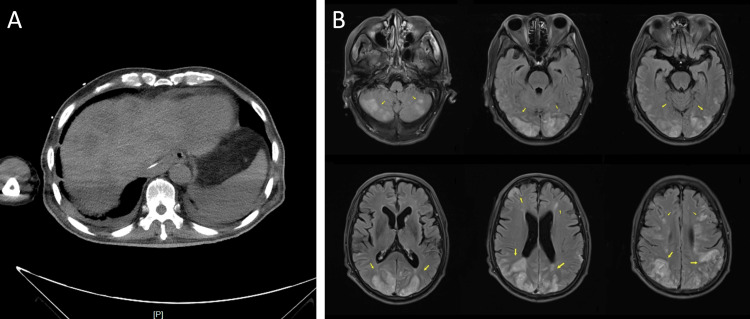
Imaging manifestations of the patient A: Abdominal CT showed multiple low-density shadows in the liver; B: MR images demonstrated extensive bilateral symmetrical lesions involving both infra- and supratentorial structures

A 63-year-old male with a prior diagnosis of cardia cancer underwent radical tumor resection, and two months later, he developed hepatocellular carcinoma metastases (Figure 1A). The patient experienced sudden-onset unconsciousness and remained unconscious without obvious causes three months after being diagnosed with hepatocellular carcinoma. Physical examination showed: body temperature: 36.9 °C; blood pressure: 125/92 mmHg; blood oxygen saturation: 97%. Blood gas analysis showed: pH: 7.45; pO2: 81 mmHg; partial pressure of carbon dioxide (pC02): 39 mmHg; bicarbonate (HCO3-): 27. 1 mmol/L; base excess blood (BE(B)): 3.0 mmol/L; K: 2.5 mmol/L; Na: 142 mmol/L; Ca: 1. 06 mmol/L; glucose (GLU): 10.4 mmol/L; Lac:1.3 mmol/L. A coagulation test showed an international normalized ratio (INR) of 1.21 and activated partial thromboplastin time (APTT) of 27.3 s. Routine blood tests and CRP were roughly within normal range. Aspartate aminotransferase was 88 U/L. No obvious abnormalities were found in renal function, electrolytes, bilirubin series, amylase, myocardial injury markers, and procalcitonin. Neuroimaging findings (Figure 1B) demonstrated widespread lesions in the frontal, temporal, parietal, and occipital lobes of the bilateral cerebral, including cortical and subcortical white matter and supratentorial and infratentorial regions of the cerebellum, involving the bilateral anterior and posterior circulations.

**Figure 2:**
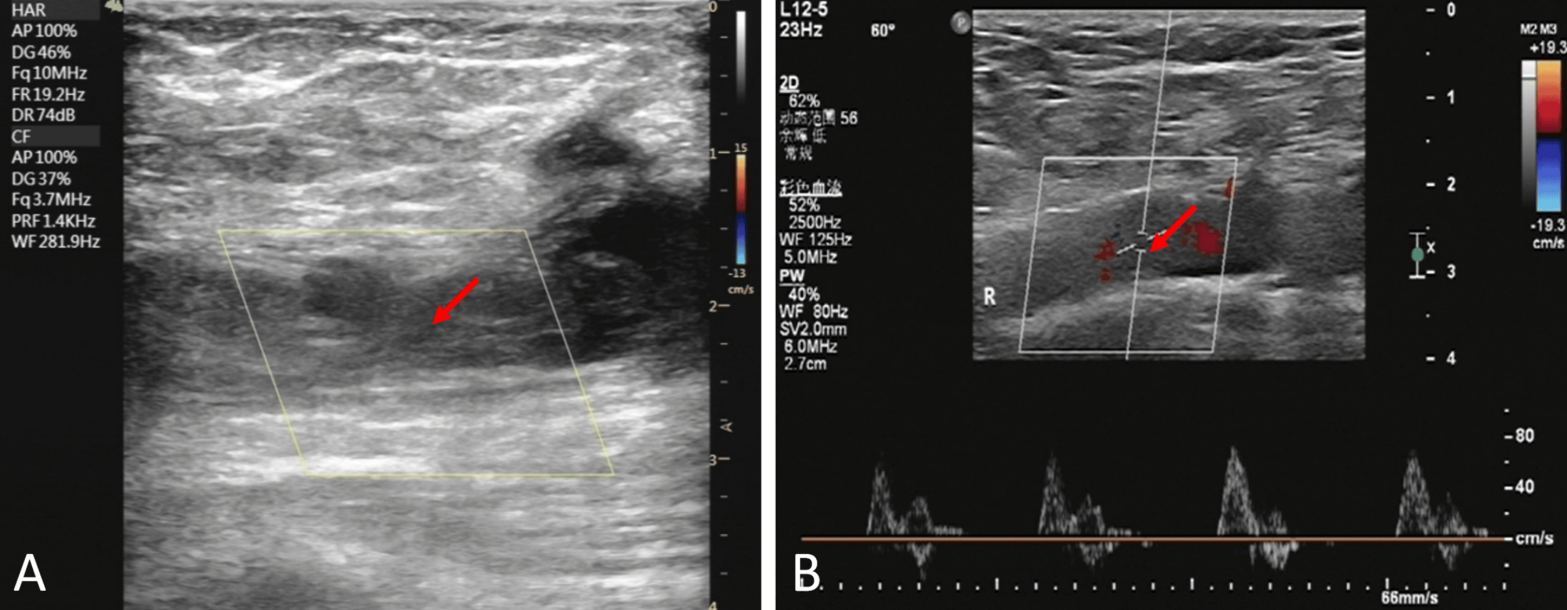
Color Doppler ultrasound A: Venous thrombosis in the right femoral vein; B: Heterogeneous atherosclerotic plaques

Echocardiography showed segmental solid wall motion abnormalities and echogenicity at the left ventricular apex, suggesting thrombus formation. Concurrent vascular evaluation identified multifocal thrombosis: Color Doppler ultrasound demonstrated venous thrombosis in the right femoral vein (Figure 2A) and heterogeneous atherosclerotic plaques in bilateral superficial femoral arteries (Figure 2B). Echocardiography showed segmental solid wall motion abnormalities and echogenicity at the left ventricular apex, suggesting thrombus formation. Laboratory workup showed markedly elevated D-dimer levels (3.740 μg/mL), exceeding the diagnostic threshold of 2.0 μg/mL associated with 82.9% specificity for TS.

**Figure 3:**
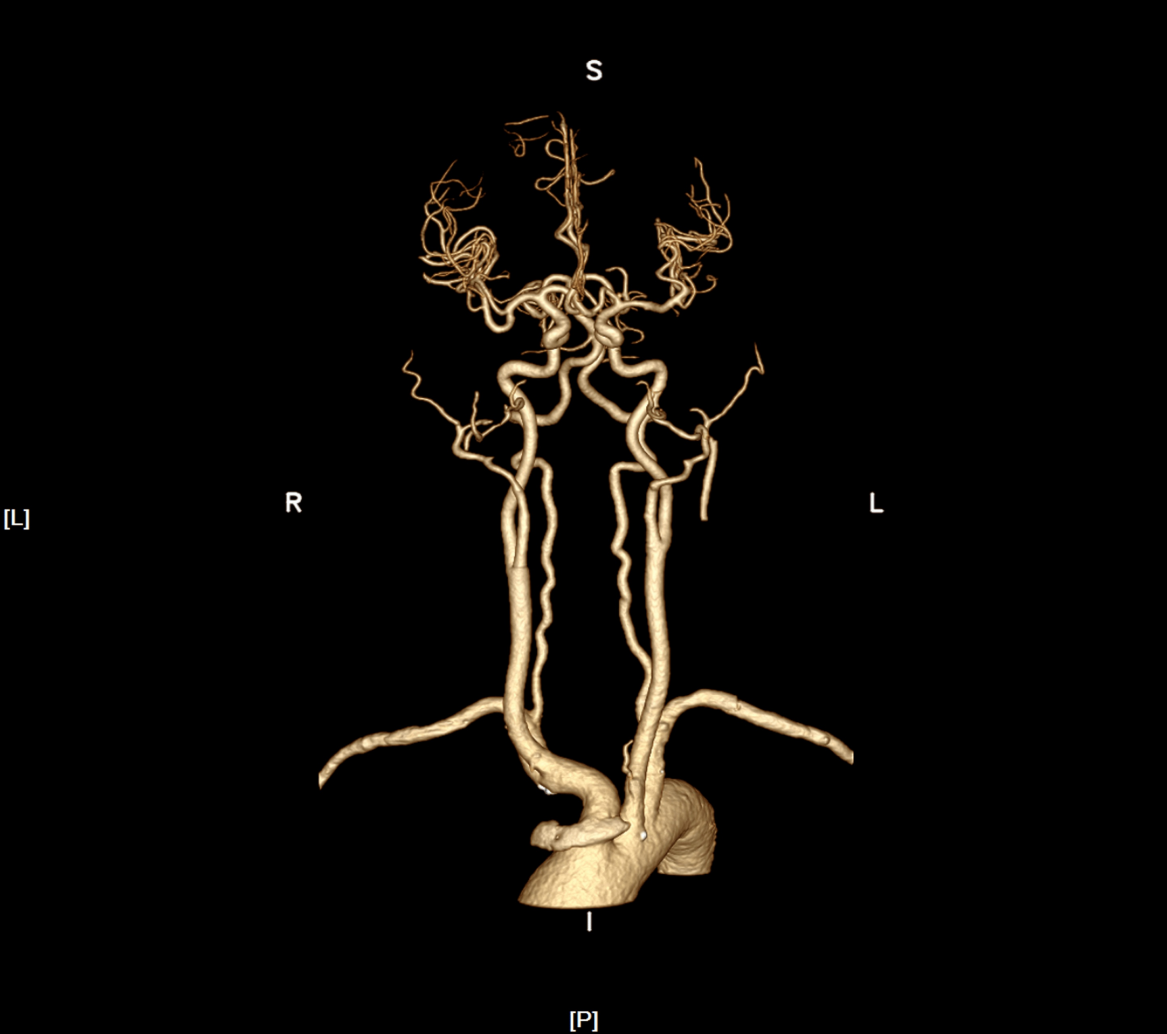
CTA of the head and neck CTA: computed tomography angiography

Computed tomography angiography (CTA) of the head and neck (Figure 3) did not demonstrate any significant stenosis or occlusion, thereby ruling out a diagnosis of atherosclerotic plaque-type cerebrovascular disease. Additionally, enhanced imaging of the brain parenchyma did not exhibit any notable enhancement, which is not characteristic of tumor metastasis. There was no indication of cardiogenic thrombosis, such as atrial fibrillation or rheumatic heart disease, indicating that the clinical presentation was not consistent with cardiogenic cerebral embolism. Combined with the patient's multifocal cerebral infarction, multiple arteriovenous thrombosis, tumor history, and D-dimer level, he was diagnosed with TS.

After the diagnosis of TS, the patient was initiated on a dual antithrombotic regimen consisting of continuous intravenous nadroparin infusion (2 mL, three times daily) co-administered with aspirin 100 mg daily. Serial laboratory monitoring revealed a progressive elevation of D-dimer levels from baseline 3.74 μg/mL to 4.18 μg/mL over 72 hours, and APTT levels remained less than 40 s, suggesting subtherapeutic anticoagulation. This clinical course was complicated by acute ST-segment elevation myocardial infarction manifesting as characteristic ECG changes.

**Figure 4:**
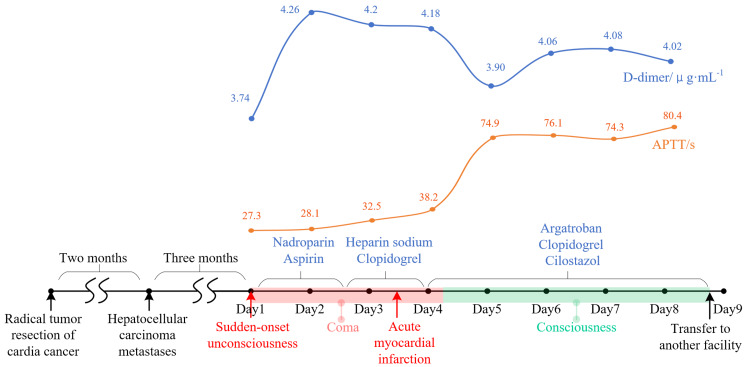
The timeline of the treatment process

Then, the antithrombotic strategy was systematically optimized: 1) Nadroparin was discontinued in favor of argatroban infusion (30 mg, twice daily) to achieve more predictable anticoagulation; 2) Aspirin was transitioned to clopidogrel 75 mg daily; 3) Cilostazol 50 mg twice daily was added to establish antiplatelet therapy. Post-intervention monitoring demonstrated (Figure 4): a) No subsequent thromboembolic events; b) Reduction in D-dimer levels (4.26 → 4.02 μg/mL); c) APTT levels of about 70-80 s; d)Negative occult blood in urine and stool, no subcutaneous and gingival bleeding was observed. Despite these therapeutic gains, the patient remained surgically ineligible due to multifocal cerebral infarctions on cranial CT and prohibitively high anesthetic risk (American Society of Anesthesiologists (ASA) class IV). At the request of his family, a palliative transfer was arranged to another facility.

## Discussion

TS is characterized by a distinct temporal association between malignancy and thrombotic events, most notably ischemic stroke. Clinical manifestations often extend beyond cerebrovascular involvement, such as developing synchronous thrombotic events, including arterial/venous thromboembolism, pulmonary embolism, and non-bacterial thrombotic endocarditis, particularly in adenocarcinomas of pulmonary or gastric origin [3].

The pathophysiology, though not fully elucidated, involves tumor-mediated disruption of coagulation homeostasis through multifactorial mechanisms. Malignant cells lead to a hypercoagulable state via endothelial injury, tumor-derived mucins, platelet activation, and excessive release of tissue factor (TF)-bearing micro-particles procoagulant vesicles demonstrated to increase thrombin generation [1].

TS lacks universally accepted diagnostic criteria but exhibits distinctive neuroimaging correlates. When MRI reveals lesions affecting the bilateral cerebral and cerebellar cortex, as well as subcortical or deep white matter regions, and these lesions are characterized as non-enhancing, non-ring-like clustered or spot-like, TS should be considered. This consideration should be further justified when coupled with a clinical history of tumors, synchronous macrovascular thrombosis (venous/arterial), and elevated levels of D-dimer and tumor markers [3]. D-dimer serves as a key biological marker. A D-dimer level of 2.0 μg/mL can achieve a specificity of 82.9% for the diagnosis of TS, and its level is strongly correlated with the severity of the patient's hypercoagulable state, the response to treatment, and the potential risk of mortality [4].

There are no established clinical rules available for TS. Guidelines recommend that patients experiencing cancer-associated thrombosis during the active phase of their malignancy should receive anticoagulation therapy for a duration exceeding six months, with LMWH being preferred [5]. Thrombolysis and mechanical thrombectomy could be considered when necessary [6]. Additionally, if the patient's clinical condition allows, active treatment of the underlying malignancy should also be pursued [7].

## Conclusions

We report TS-associated extensive multifocal cerebral infarctions and the triple-pathway anticoagulation-antiplatelet salvage therapy after LMWH resistance. This case offers a potential management paradigm for refractory hypercoagulable states, but the triple-pathway therapy carries high hemorrhagic risks, where coagulation indicators should be closely monitored. The pathophysiology and diagnostic criteria of TS remain unclear, and clinical rules still rely on physician discretion and clinical experience, where further high-quality investigations and studies between coagulation and tumors are needed to form a clear and unambiguous consensus policy.
